# An Unusual Complication of Suprapubic Catheter Insertion

**DOI:** 10.1100/tsw.2006.378

**Published:** 2006-05-25

**Authors:** Krishnan Ananthakrishnan, Rajinikanth Ayyathurai, J.K. Chiran, Waliul Islam, Vaikuntam Srinivasan

**Affiliations:** Department of Urology, Glan Clwyd Hospital, Sarn Lane, Rhyl, UK

**Keywords:** suprapubic catheter, cystoscopy, small bowel obstruction, mesenteric injury

## Abstract

A patient who had a small bowel mesentery perforation following insertion of a suprapubic catheter (SPC) is described. He had no bowel complaints immediately following the procedure, but presented 10 weeks later with insidious onset bowel obstruction due to the kink caused by the catheter. This complication occurred despite cystoscopy control and adequate bladder distension prior to the procedure. This isolated case illustrates the fact that regardless of the ease and frequency of SPC insertion, complications do occur.

## INTRODUCTION

Suprapubic bladder drainage is considered a valid and useful adjunct in various scenarios. Most commonly in urology practice, it is invaluable in patients with long-term catheters (due to various causes) and in postoperative situations. Numerous reports have emphasized the decreased bacteriuria, decreased hematuria, ease of change of catheter, and in young ones, the social aspect of the ability to get on with one's life (sexual needs, etc.) and safety. Complications do occur with suprapubic catheter (SPC) insertion and are mostly limited to mild hematuria, localized cellulitis, and catheter malfunction. Very rarely; major complications do occur after suprapubic cystostomy. Through and through perforation of the bladder, severe hematuria, and bowel perforation *per se* have been reported. The differential diagnosis of acute abdomen in a patient with a recent SPC insertion is a possible misplacement of the device. For the first time in the literature, we report a case of mesenteric perforation and, thus, small bowel obstruction caused by SPC insertion. We also discuss the anatomical variants that could have led to this complication.

## CASE PRESENTATION

An 83-year-old man known to have carcinoma of the prostate and on clean intermittent self-catheterization, had SPC inserted for ease of lower urinary tract management. His prostatic carcinoma was well controlled with hormonal manipulation. Rigid cystoscopy showed large-capacity trabeculated bladder. He had not had any previous abdominal surgery. The SPC was placed (16 F two-way Foley) using Bards® suprapubic catheterization set under general anesthetic with cystoscopic control and without any difficulty.

In the early postoperative period, he developed a hypotensive episode and complained of generalized abdominal discomfort and pain. Radiological investigations such as X-ray abdomen and erect chest film showed no abnormality. Bowel sounds were normal with localized tenderness around the SPC site and hypogastric region. Hemoglobin dropped from 11.4–7.6 gm/dl in the ensuing 48 h. He was treated conservatively and received 3 units of blood transfusion.

The SPC functioned satisfactorily until 10 weeks after insertion, when he presented with nausea, vomiting, lower abdominal pain, and distension. The SPC drained clear urine. Clinical and radiological evidence pointed to small bowel obstruction. Laparotomy revealed an intraperitoneal SPC passing via small bowel loop mesentery and into the bladder (Fig. 1). There was no evidence of any adhesions on the dome of the bladder, which could have caused this complication. The intraperitoneal part of the SPC acted as a band and kinked the small bowel loop, thus causing small bowel obstruction. The SPC was removed and replaced. The hole in the mesentery was closed. The patient recovered well with no other complications.

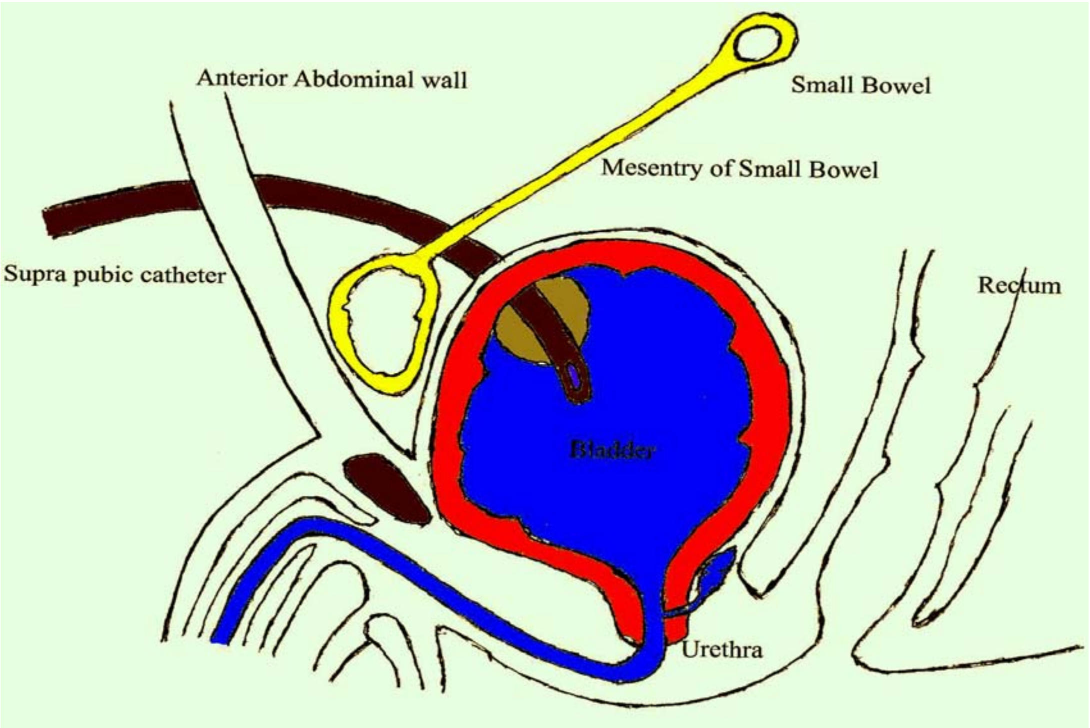

**FIGURE 1.** Showing a suprapubic catheter perforating the mesentery and going into the bladder.

## DISCUSSION

This case illustrated the potential morbidity associated with a commonly practiced procedure. SPC insertion, being a blind invasive procedure, is prone to cause complications such as bowel injury, bowel obstruction, and hemorrhage. To our knowledge, this is the first reported case of mesenteric perforation leading to small bowel obstruction.

Generally, SPC insertion is done under local anesthesia in an emergency setting. Surgeons prefer to do this as an elective procedure under general or regional anesthesia if it is done for long-term urinary drainage. This gives the opportunity to perform cystoscopy and reduces discomfort to the patient. It also enables the surgeon to place the catheter under vision and minimize complications. In most situations, the bladder distends sufficiently to push the peritoneum and its contents, thereby enabling safe extraperitoneal puncture through the mid-line (Linea Alba). However, iatrogenic bowel injuries have been reported[1,2]. These injuries happen when the bladder is nondistensible, in the presence of deep peritoneovesical fold (anatomic variant)[3] and previous lower abdominal surgery, leading to loops of bowel adherent to the bladder and anterior abdominal wall. To some extent, the deep peritoneovesical fold could be obliterated by placing the patient in the Trendlenberg position, in addition to following the manufacturer's instructions for SPC placement. If difficulty is encountered with distension such as a small-capacity bladder or in an obese patient, Hay-Groves dilators, which have been customized by drilling a hole 0.5 cm from the tip, can be used to tent up the bladder onto the anterior abdominal wall, especially in women. In men, the SPC should be placed with an open cystotomy in the above situations. The adhesions associated with the previous abdominal surgery could be avoided with laparoscopic assistance.

This case demonstrates the element of serious risk associated with the commonly practiced SPC insertion. Strict adherence to adequate bladder distension minimizes the risk of intraperitoneal puncture, but does not alleviate it completely.
